# Socioeconomic disparities in children’s neurodevelopment before and during primary education: Evidence from Bagamoyo, Tanzania

**DOI:** 10.1371/journal.pone.0354139

**Published:** 2026-07-27

**Authors:** Msilikale Walter Manyiri, Georg Loss, Susanne P. Martin-Herz, Deborah Sumari, Nicolaus Gutapaka, Thabit Athuman, Sylvester Simando, Sylvia Jebiwott, Xue Wu, Karim Manji, Ally Olotu, Michelle S. Hsiang, Günther Fink

**Affiliations:** 1 Household Economics and Health System Research Unit, Department of Epidemiology & Public Health, Swiss Tropical and Public Health Institute, Kreuzstrasse 2, Allschwil, Switzerland; 2 University of Basel, Klingelbergstrasse 50, Basel, Switzerland; 3 Biomedical Research and Clinical Trials Department, Ifakara Health Institute, Mikocheni, Kinondoni, Dar es Salaam, Tanzania; 4 Department of Biostatistics, Epidemiology and Behavioural Sciences, School of Public Health, Catholic University of Health and Allied Sciences, Bugando, Mwanza, Tanzania; 5 Division of Developmental Medicine, Department of Pediatrics, University of California San Francisco (UCSF), San Francisco, California, United States of America; 6 Malaria Elimination Initiative, Institute of Global Health Sciences, University of California San Francisco, San Francisco, California, United States of America; 7 Department of Pediatrics and Child Health, School of Medicine, Muhimbili University of Health and Allied Sciences, Upanga, Dar Es Salaam, Tanzania; 8 Department of Pediatrics, University of California San Francisco, Benioff Children’s Hospital, San Francisco, San Francisco, United States of America; 9 Department of Epidemiology and Biostatistics, University of California San Francisco, Mission Hall: Global Health & Clinical Sciences Building, San Francisco, California, United States of America; Chittagong Medical College, BANGLADESH

## Abstract

**Background:**

Socioeconomic disparities in children’s neurodevelopment constitute a critical yet understudied developmental challenge in low- and middle-income countries (LMICs), yet longitudinal evidence from Sub-Saharan Africa (SSA) remains limited. This study aimed to evaluate the relationship between household wealth and neurocognitive trajectories in a cohort of Tanzanian children aged 6 months to 10 years.

**Methods:**

We randomly selected 590 children aged 6 months to 10 years in Bagamoyo district, a low to moderate-malaria-endemic area in Tanzania. Participants completed comprehensive, age-appropriate neurodevelopmental assessments. The family’s socioeconomic status was derived from their household assets. Standardized, age-adjusted neurodevelopmental scores were regressed on socioeconomic status tertiles and by developmental periods: early childhood (6 months-3 years), preschool (4–5 years), and school age (6–10 years).

**Results:**

While children from high- and low-Socioeconomic status (SES) families start with similar neurodevelopmental scores, a significant and sustained gap begins to emerge around age 2 years. This divergence results in a substantial disadvantage for children from the poorest households, with scores showing a difference of −0.36 Standard Deviation (SD) (95% CI: −0.64 to −0.08) during the preschool years and −0.41 SD (95% CI: −0.73 to −0.09) at school age.

**Conclusion:**

Our results suggest that programs targeting the preschool period may be most effective in mitigating disparities in neurodevelopment.

## 1. Introduction

The strong empirical relationship between parents’ socioeconomic status (SES) and child development has been well documented. SES profoundly influences a child’s development across physical, mental, and health domains, acting as a multidimensional determinant of developmental, academic, health, and socioeconomic outcomes from early childhood through adolescence [1–7]. Children from lower SES families are more likely to have developmental delays and experience worse health, educational, and labor market outcomes compared to children from better-off households [8–11]. SES gradients in neurodevelopment emerge early in life and can be observed across various domains, including language, memory, and executive functions, as well as brain structure and function [8]. These disparities emerge through a complex set of pathways involving genetics, stress, cognitive stimulation, parenting, nutrition, and other environmental factors [1,8,12].

### 1.1. The SES gradient across high- and low-income settings

Evidence quantifying the magnitude of these gradients has emerged primarily from high-income settings, where large longitudinal cohorts have made it possible to isolate and track their developmental consequences. In the United States, studies using large cohorts, like those funded by the National Institutes of Health (NIH), have shown that by the time they start school, children from lower SES backgrounds score lower on standardized cognitive tests than their peers from higher SES backgrounds [13]. These disparities are particularly notable in domains such as language and executive functions, with evidence suggesting that these gaps begin to emerge as early as 18 months of age [14]. Research also indicates that much of this gap growth occurs in early childhood, prior to school entry [15].

Likewise, cognitive and socioemotional development across the first ten years of life have been verified by studies from the UK Millennium Cohort Study and the Avon Longitudinal Study of Parents and Children (ALSPAC) [16,17]. Converging evidence from low- and middle-income countries (LMICs) has confirmed that these gradients are not confined to high-income settings alone. Multi-country analyses, including data from Bolivia and South Africa have documented that household SES predicts cognitive and language development from infancy through mid-childhood across highly diverse cultural and economic contexts, although with variation in magnitude across settings [18,19]. This reinforces that the link between lower developmental potential and family SES is a universal, cross-culturally consistent phenomena rather than a product of any particular environment.

### 1.2. Developmental timing and the home environment

Beyond establishing the universality of these gradients, literature has increasingly sought to identify the specific developmental windows during which SES-linked disparities first take root. Each childhood period presents different opportunities and challenges [20]. The early childhood years are vital for neurodevelopment, particularly for the formation of language and memory skills. Disparities in these skills between socioeconomic groups are well documented and generally emerge relatively early, typically by the second year of life [21]. Research underlines the importance of the home environment during this period; stable, rich home literacy environments (HLEs) and stimulating parent-child interactions significantly predict better future language and cognitive abilities [21–23].

Noble et al. established that HLE quality moderated the relationship between household socioeconomic status and newborn language development scores in the United States, indicating that the home environment is the primary mechanism by which socioeconomic disadvantage influences early neurodevelopment.[14]. Evidence from LMICs corroborate this conclusion: a prospective cohort study in rural Bangladesh revealed that caregiver stimulation and home learning resources accounted for a significant percentage of the SES gradients in language and cognitive scores. [24]. Similarly a study conducted in South Africa identified HLE quality as a key pathway through which household poverty translates into poorer early cognitive development [25]. However, maternal psychosocial factors such as depression and intimate partner violence have been shown to affect early childhood cognitive and behavioral development negatively [26–28].

### 1.3. Schooling, fadeout, and the persistence of disparities

Home-based influences, however, do not operate in isolation; as children approach school age, new environmental inputs begin to reshape their developmental trajectories.. High-quality preschool/kindergarten and primary school experiences can stimulate cognitive and academic achievement, potentially helping children catch up even if they displayed earlier delays [10,20,29,30]. In Jamaica, the Responsive Stimulation intervention, delivered to stunted infants aged 9–24 months produced sustained cognitive and psychosocial gains that persisted into adulthood [31]. However, entering a low-quality school or persistently experiencing low SES-related stress can also aggravate disparities [8,20]. In addition, early intervention gains in neurodevelopment may diminish without sustained support or quality follow-up, mainly for children with high cumulative risks [20,32]. This fadeout of early intervention effects is well documented in evaluations of the US Head Start program, where cognitive gains observed at kindergarten entry were substantially attenuated by Grade 3 [33]. Thus, while schooling offers recovery opportunities, structural inequities may hinder its compensatory effects, hence requiring multidisciplinary, sustained interventions targeting cognitive skills, executive functions, nutrition, and supportive home or school environments [34,35]. Whether the transition to formal schooling moderates SES-related neurodevelopmental trajectories in SSA contexts, and whether any such moderation is sufficient to alleviate the gradients established in early childhood, remains a critically under-examined question that this study addresses.

### 1.4. The sub-Saharan African and Tanzanian context

Although extensive research has explored the link between SES and neurodevelopment in high-income settings [1,8,11,36,37], a significant gap remains in understanding this relationship within low- and middle-income countries (LMICs). Sub-Saharan Africa (SSA) specifically bears the highest burden of children not reaching their developmental potential. This is largely due to unique contextual challenges like poverty, malnutrition, prevalent infections such as Human Immunodeficiency Virus (HIV) and malaria, violence, rapid socio-cultural shifts, and limited access to early childhood care and education [38–43], With each of these risk challenges carrying its own neurodevelopmental consequences. Tanzania specifically exemplifies a socioeconomic context that is marked by high disease burdens (e.g., HIV, malaria) and systemic inequities. Latest estimates suggest that 58% of children live below the poverty line, with restricted access to healthcare, education, a nutritious diet, and other essential resources [44–48].

While the negative impact of low SES background on neurodevelopmental outcomes seems well established, the magnitude of these differences seems to vary widely within SSA [41,45,49]. These differences are likely due to both differences in population and settings and differences in the neurodevelopmental instruments and outcome domain constructs used [34,41]. Data to compare children at different ages and at different developmental stages is scarce [34,41,49,50]. This measurement heterogeneity is a primary driver of the inconsistent and often incomparable findings across SSA studies and represents a major barrier to understanding how SES shapes neurodevelopment across distinct developmental stages.

Despite considerable success in adapting neurodevelopmental tools like the Bayley Scales of Infant and Toddler Development to specific countries and new tools directly developed in LMICs, such as the Malawi Development Assessment Tool (MDAT) and the Caregiver-Reported Early Development Instruments (CREDI), many local studies still rely on instruments that lack validation against the population’s linguistic and social realities [45,46,51,52]. The present study addresses this methodological gap by employing neurodevelopmental instruments that have undergone validation against the linguistic, cultural, and socioeconomic realities of the specific Tanzanian population under study [53].

### 1.5. Evidence gaps and the present study

Altogether, the literature reveals key gaps this study addresses. First, while SES–neurodevelopment associations in SSA are mostly cross-sectional, no Tanzanian study has examined how these evolve continuously from infancy to middle childhood, limiting inference on whether disparities widen, narrow, or persist. Second, although formal schooling is theorized to moderate SES-related disparities, this has not been tested in Tanzanian or East African community samples. Third, inconsistent SSA findings reflect measurement heterogeneity; this study uses locally validated neurodevelopmental tools, enabling valid cross-age comparisons and advancing regional evidence.This study explores the relationship between household wealth and childhood neurodevelopment in children aged 6 months to 10 years from Tanzania using instruments previously validated in this setting [53]. In doing so, we make two important contributions to the SSA neurodevelopmental literature: (1) characterizing, for the first time in this setting, how SES-related neurodevelopmental disparities manifest and evolve across three distinct developmental stages, i.e., early childhood, pre-school age, and school age; and (2) we test whether access to formal schooling moderates SES-related neurodevelopmental trajectories, providing evidence relevant to both developmental theory and educational policy in Tanzania and comparable LMIC contexts.

## 2. Methods

### 2.1. Study design and setting

This cross-sectional analysis leveraged baseline data from the Child Health and Infection with Low-Density Malaria (CHILD) randomized intervention study (ClinicalTrials.gov: NCT05567016; PACTR202311470737025). This parent trial investigates the long-term health and socioeconomic impacts of detecting and treating low-density malaria infections in children [54]. In the parent trial six hundred children aged 6 months to 10 years from Kiwangwa and Fukayosi wards in Tanzania’s Bagamoyo district were enrolled. The present analysis focused on baseline socioeconomic and neurodevelopmental data collected before trial interventions.

### 2.2. Participant selection and randomization

#### 2.2.1. Participants selection.

**Fig 1:**
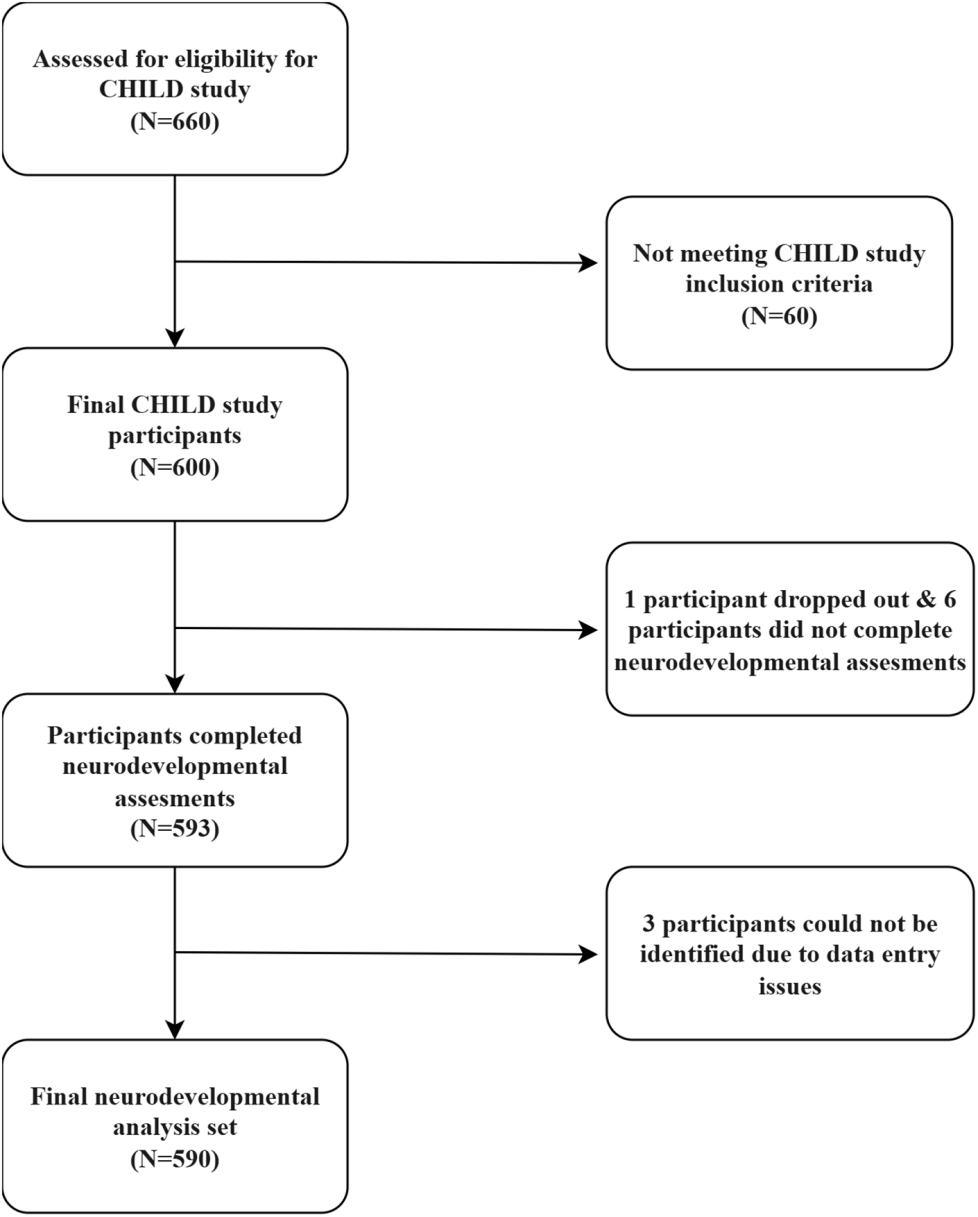
A study flow chart. Out of the 600 enrolled participants, seven were dropped due to incomplete neurodevelopmental assessments, and three were excluded due to data entry issues, leaving a final analysis set of 590 participants.

We created household listings in the Kiwangwa and Fukayosi wards. From these listings, we randomly selected 660 children aged 6 months to 10 years to be invited for screening (Fig 1). To avoid data clustering and limit potential intervention contamination within families, we restricted eligibility to only one child per household. Initial screening and informed consent took place during home visits. Children were eligible if they were local residents, were not currently enrolled in other trials, and if their guardians agreed to seek care at the study clinic for illnesses and avoid outside medications, including herbal remedies [54].

We excluded children with a history of stage 3 or 4 HIV/AIDS, diabetes, or cancer, as well as those with a known allergy to artemether-lumefantrine (AL) or a weight below 5 kg [54]. Following the household screening, 600 eligible children were enrolled. Enrollment was finalized at the study health facility, where baseline assessments were conducted and participants were assigned to study arms. Participants were required to remain in the study for two years, though they could be withdrawn in cases of severe adverse events, loss to follow-up, or non-compliance with study protocols.

#### 2.2.2. Randomization.

Participants were allocated to one of three study arms in a 1:1:1 ratio (n = 200 per arm). To ensure the groups were balanced, we used a computer-generated randomization sequence stratified through age category (6 months −5 years; 6–10 years), geographical location, and gender. We use the minimization method [55], to maintain balance in baseline characteristics as enrollment continued. To prevent bias, this procedure was overseen by a team member who was not directly engaged in the study’s execution. While the trial was open label for participants and clinicians, the laboratory staff and data analysts remained blinded to the intervention assignments throughout the study. To minimize bias, primary outcome assessments were standardized through annual staff training and the use of identical case record forms across all study arms [54]. Of the 600 enrolled participants, 1 withdrew and 6 did not complete the neurodevelopmental assessments. Three more participants were excluded due to ambiguous identification resulting from data entry errors. This left a final set of 590 participants for the neurocognitive analysis.

### 2.3. Consent procedures

Trained study staff obtained written informed consent from the parents or guardians of all potential participants before the commencement of any study procedures. The written consent forms, which included a statement about the possibility of future testing of collected samples, were provided in Swahili, the national language. Before the signing took place, staff verbally assessed the comprehension of the study details by the parents or guardians. In accordance with local guidelines, written minor assents were not obtained, as all study participants were under 13 years of age during the study period, which is the age threshold below which written assent is not standard practice.

### 2.4. Data collection procedures

All baseline assessments and interviews relevant to this analysis took place between 18 July and 29 November, 2023. Information was collected directly during neurodevelopmental assessments at the Kiwangwa or Fukayosi study clinics, and parent interviews at the clinics or during home visits. Doctors and nurses with significant training in delivering the neurodevelopmental batteries as outlined by Loss et al. conducted all examinations and data collecting [53]. The Open Data Kit (ODK) program was used to digitally gather data on Android-based tablets [56]. Castor Electronic Data Capture (EDC) was used to collect clinical data at the clinics located at the study sites. EDC is a web-based data capture system with real-time validation to ensure data quality and consistency [57]. To ensure continued adherence to research protocols,the study coordinator regularly supervised all study personnel.

### 2.5. Variables

We gathered demographic information on age, sex, village of residence, school information (ever attending school or not) and then grouped into childhood periods; early childhood (0.5–3 years), preschool age (4–5 years), and school age (6–10 years) as children are legally expected to begin Standard one of primary school at age 6 in Tanzania [58]. Socioeconomic information on presence or absence of the following household assets: electricity, ownership latrine/flush toilet, radio, TV, refrigerator, gas/electric stove, generator, bed(s), lamp(s), oven, hoe(s), sewing machine, motorbike, car, satellite dish, mobile phone, smartphone, and finished floors was also collected. Socioeconomic status (SES) was then assessed using a wealth index derived from Principal Component Analysis (PCA) of the household assets. Binary variables for each asset were included in the PCA to generate a composite factor score. Households were then categorized into tertiles (low, middle, and high SES) based on these scores to serve as a proxy for long-term household socioeconomic status.

Neurodevelopment was evaluated using age-appropriate instruments that assess core aspects of cognitive ability, which had been validated locally, specifically within the CHILD study context [53]. For children aged 6 months to <3.5 years, we used the standardized Global Scales for Early Development (GSED). The GSED was designed to objectively measure the motor, language, cognitive, and social-emotional development of children 0–3 years in low- and middle-income countries [59]. We administered the directly observed Long Form (GSED-LF), which has been validated in Tanzania. Assessments were conducted in Kiswahili, using the Tanzania-adapted version, at the Kiwangwa and Fukayosi dispensaries, by trained health professionals who had completed the standard GSED training protocol. Each child’s exact age was confirmed from their clinic card so that the tablet application could select the age-appropriate item set/ start point, and the caregiver was present throughout. Each assessment was conducted in a quiet, distraction-free space, with all materials prepared before testing began. In line with the standardized GSED procedure, assessors first established rapport with the child and caregiver, presented the tasks in a playful, low-pressure manner with consistent encouragement and breaks as needed, and asked caregivers not to prompt the child during testing. Responses were recorded directly on the GSED tablet application, which selects age-appropriate items and minimizes transcription errors, with assessments conducted under ongoing supervision to maintain consistency across assessors.

For children aged 3.5 to 5 years inclusive, we used the International Development and Early Learning Assessment (IDELA) battery, which directly assessed motor development, emergent literacy, emergent numeracy, and social-emotional development in preschool-aged children [60]. The following general intelligence and executive functioning constructs of the East Africa Cognitive Assessment Battery (EACAB) [61] were used to assess all school-aged study children (i.e., 6–10 years), except where otherwise stated: learning (6–8 years inclusive), planning, sequential processing, and two working memory constructs. In addition, school-aged children were tested for sustained attention using the pencil tap test (6–8 years inclusive) [62] and the code transmission sub-test of the Test of Everyday Attention for Children battery (TEA-Ch) [63].

All neurodevelopmental instruments were administered individually in a standardized, quiet setting by trained assessors. Administration times were approximately 10–25 minutes for GSED, 21–43 minutes for IDELA, and 45–60 minutes for the EACAB battery. To ensure data quality and minimize child fatigue, assessments were conducted in a single session with scheduled brief intervals between sub-tests.

GSED and IDELA instruments, by definition, provided a single overall functional score for each participant, which we standardized (z-scores) within each instrument for subsequent analysis. For the school aged children we generated composite scores using all domain tests available at the respective test age ranges (i.e., two test batteries for (i) 6–8 year olds: learning, planning, sequential processing, executive functioning, and sustained attention, and (ii) 9–10 year olds: planning, sequential processing, executive functioning, and sustained attention). We then used principal component analysis for the two age group batteries to derive the respective first principal component and generate standardized z-scores within each age battery. To derive the final global cognitive scores, results were pooled across all 50 iterations. The first principal component (PC1) explained an average of 49.17% of the variance in the 6–8years battery and 41.83% of the variance in the 9–10years battery. In both cases, the factor structure was highly stable across imputations, as evidenced by the negligible standard deviations in factor loadings. To adjust for the natural age-related progression of neurodevelopment, we predicted an age-residualized neurodevelopment score from a linear regression model entering age in years.

While tests were designed to minimize refusal, we found some refusals for sustained attention and executive function tests, especially at younger ages within the instrument group (refusals for 6–8 year olds: Pencil tap test: 7%, executive function working memory test 1: 8.21%, executive function working memory test 2: 19.%; refusals for 9–10 year olds: sustained attention test: 7.12%). Missing information on these sub-tests was filled in using the iterative Markov chain Monte Carlo multiple imputation method (using 50 imputed sets with Stata 18’s mi package). Specifically, we employed Multiple Imputation by Chained Equations (MICE) to generate 50 imputed datasets. Principal Component Analysis (PCA) was conducted separately within each of the 50 imputed datasets using a correlation matrix. The resulting first principal component (PC1) scores were then averaged across imputations to produce a single pooled estimate for each participant, which was subsequently z-score standardized for analysis.

### 2.6. Statistical analysis

We summarized characteristics of the study participants by counts and percentages or means and standard deviations as appropriate and used Student’s t-test or chi square test to test for differences between socioeconomic strata. SES of participants’ households was based on a wealth index, which was derived as the first principal component of available household assets [64–66]. Families were then grouped by their index into households with low (tertile 1), middle (tertile 2) or high (tertile 3) SES. To measure socioeconomic differences related to transitions into the formal schooling system, we calculated the proportions in school at ages 6−8 and kernel-weighted local polynomial regression analysis to visually explore non-linear relationships over age. We then explored the relationship of age‐residualized neurodevelopment scores and the extreme socioeconomic strata (high vs. low tertile) using local polynomial regressions and mean differences at each age-year group. Finally, to examine whether the association between SES groups and age-residualized neurodevelopment scores varied by childhood periods, early childhood (6 months −3 years), preschool age (4–5 years), and school age (6–10 years), we estimated a linear regression model adding a multiplicative interaction term for SES tertiles and childhood periods. We additionally added enumerator fixed effects to account for systematic variations in test administration.

Our primary objective was to document the magnitude of socioeconomic gradients. Thus, we included interviewers fixed effects in our regressions to control for potential observer differences but did not adjust for other factors (such as caregiver education) which might mediate the relationship between socioeconomic status and child development. We report main effects representing differences in residualized cognitive scores relative to individuals with high childhood SES. Analyses were performed using STATA 18 [67]. P-values of <0.05 were considered statistically significant, and 95% confidence intervals were reported. The complete analytical code is provided in S1 Text, and the de-identified minimal dataset is available in S1 Data.

### 2.7. Ethics statement

The study protocol has been reviewed and approved by Institutional Review Boards at University of California, San Francisco (Reference#: 342371), and Ifakara Health Institute, Tanzania (Reference#: IHI/IRB/No:08–2023), and by the Tanzania National Health Research Ethics Review Committee (Reference#: NIMR/HQ/R.8a/Vol.IX/4204). The study was approved by the President’s Office, Regional Administration and Local Government, Tanzania (PO-RALG) (Reference#: AB. 307/223/01). Additional information regarding ethical, cultural, and scientific considerations specific to inclusivity in global research is outlined in S4 File.

## 3. Results

The demographic and socio-economic characteristics of the study participants (N = 590) stratified by SES showed the overall sample was well balanced by sex, with 50.85% male and 49.15% female (Table 1). Males were overrepresented in the Lower SES group (54.50%) as compared to the Middle (47.24%) and High SES (49.49%) groups. The mean age of children was 4.36 years (Standard deviation (SD) = 2.75). Lower SES children were marginally younger on average (mean = 3.75, SD = 2.74) as compared to Middle (mean = 4.01, SD = 2.69) and Upper SES (mean = 3.85, SD = 2.91). There were no significant gender or age group differences across the SES groups.

**Table 1:** Demographic characteristics of study participants by wealth tertiles.

Variable	Category	Total		Low SES		Middle SES		High SES	
		N	%	N	%	N	%	N	%
**Total**	All children	590	100.00	231	39.15	163	27.63	196	33.22
**Sex**	Male	300	50.85	126	54.55	77	47.24	97	49.49
	Female	290	49.15	105	45.45	86	52.76	99	50.51
**Age(years)**	0	61	10.34	26	11.26	15	9.20	20	10.20
	1	110	18.64	42	18.18	27	16.56	41	20.92
	2	67	11.36	25	10.82	19	11.66	23	11.73
	3	52	8.81	25	10.82	10	6.13	17	8.67
	4	49	8.31	21	9.09	18	11.04	10	5.10
	5	65	11.02	21	9.09	20	12.27	24	12.24
	6	58	9.83	22	9.52	16	9.82	20	10.20
	7	47	7.97	20	8.66	19	11.66	8	4.08
	8	53	8.98	22	9.52	14	8.59	17	8.67
	9	27	4.58	7	3.03	5	3.07	15	7.65
	10	1	0.17	0	0.00	0	0.00	1	0.51
**Age(years)**	Mean: Standard deviation:	4.36 2.75		3.75 2.74		4.01 2.69		3.85 2.91	

This table presents a detailed breakdown of key demographic information, such as age and gender, and for the study participants, categorized by their family socioeconomic status (SES) tertile.

### 3.1. Household SES and school enrollment

**Fig 2:**
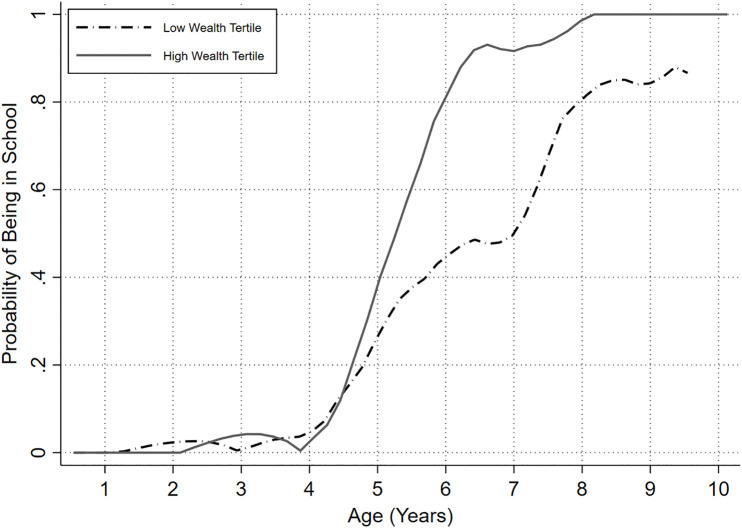
Local polynomial regression of children in school by age and wealth to explore potential enrollment effects. The figure displays the estimated school enrollment rates for children from high- and low-socioeconomic status (SES) families across different ages.

At 6 years of age, we estimate 80% of children from the high SES families were in school, while only 45% of children from low SES families were enrolled in school (Fig 2). At 7 years of age, we estimate 90% of children from the High SES were enrolled in school, while only 50% of children from low SES families were enrolled in school at that age. Similarly, at 8 years of age, almost all the children from high SES families were enrolled compared to 80% of children from low SES families.

### 3.2. Household wealth and children’s neurodevelopment

**Fig 3:**
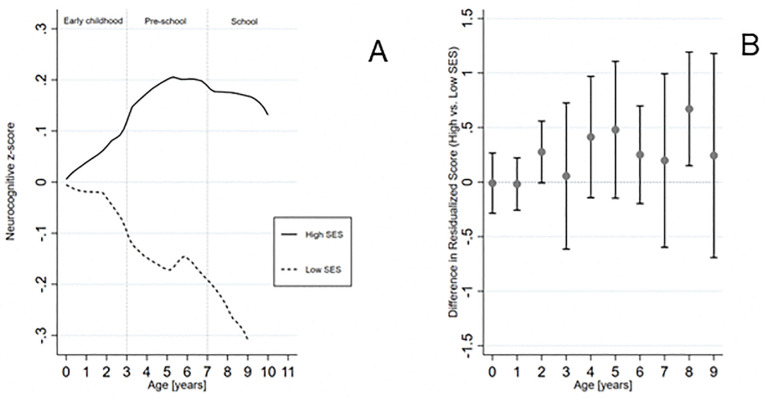
Panel A. Smoothed plot of Neurodevelopment by Socioeconomic Status (SES). This figure is a smoothed plot that illustrates the absolute neurocognitive z-scores of children from high and low socioeconomic status (SES) groups across a range of ages. It shows a clear separation in scores, with the high SES group consistently scoring higher than the low SES group. Panel B. Estimated Neurodevelopmental differences by Socioeconomic Status (SES). This figure displays the age-specific differences in neurodevelopmental scores between children from high- and low-socioeconomic status (SES) groups. The plot illustrates the estimated difference in scores at each year of age, with vertical lines representing the confidence intervals. It shows that the difference in scores becomes larger as children get older, indicating a widening gap between the two groups over time.

Children from high SES households showed higher overall mean neurodevelopmental z-scores compared to their counterparts from low SES families (difference in age-residualized average z-scores across all ages: 0.25, P-value = 0.01, see details in S1 Table). Our non-linear model revealed an age-dependent emergence of neurodevelopmental gaps between socioeconomic status (SES) groups. While neurodevelopmental scores were similar at the youngest ages, they began to diverge around age 2 years in favor of high SES highlights (Fig 3: Panel A). Comparing mean differences per age-year group showed a similar result, where around age 4, the gap between the bottom and top tertile appeared to be stabilized at a level around 0.50 standard deviations, although statistically significantly only at age 8 years (Fig 3: Panel B).

At preschool age, children from low SES backgrounds statistically significantly scored on average 0.36 SD lower (95% CI: −0.64 to −0.08) than children from high SES backgrounds. In the school age group, similarly, the difference was 0.41 SD (95% CI: −0.73 to −0.09) (Table 2). In contrast, in the early childhood (ages 6 months −3 years) group, the estimated difference was small and not statistically significant. Differences between the high and middle SES groups were much smaller and not significant for any of the three developmental periods considered (Table 2, bottom panel). The full model and its derived strata-specific neurodevelopment score averages are detailed in the Appendix (see S2 Table and S3 Table).

**Table 2:** Socioeconomic status main effects on age-residualized neurodevelopmental scores (vs. the highest SES group) by schooling.

Age-residualized neurodevelopmental score	Coefficient	95% CI Lower	95% CI Upper	P-value
**Low vs. High SES tertile**				
In Early Childhood Age	−0.02	−0.23	0.18	0.82
At Preschool Age	−0.36	−0.64	−0.08	0.01*
At School Age	−0.41	−0.73	−0.09	0.01*
**Middle vs. High SES tertile**				
In Early Childhood Age	−0.08	−0.31	0.16	0.53
At Preschool Age	−0.18	−0.47	0.10	0.22
At School Age	−0.04	−0.38	0.30	0.80

This table presents the linear regression model results, showing how socioeconomic status and developmental period, along with their interaction, are associated with neurodevelopmental scores, after adjusting for enumerators.

*P-value ≤ 0.05

Note: Linear regression model with interaction of SES and developmental period, adjusting for Enumerators.

## 4. Discussion

The principal aim of this study was to explore the relationship between household SES and childhood neurodevelopment in Tanzanian children from infancy to age 10 years. Our results show that socioeconomic disparities in neurodevelopment emerge relatively early among children in this setting. We found that disparities are concentrated mostly within the lowest tertile, who were similar to high SES children in the first three years, but then showed significant neurodevelopmental gaps during preschool (−0.36 SD) and school age (−0.41 SD). Children with low SES also showed substantially lower school enrollment at ages 6–8 years.

While neurodevelopmental score disparities among SES groups appeared less pronounced in the first three years, this finding should not be misinterpreted as an absence of need. Additionally, as children get older, their development involves more complex and interconnected skills, and a single screening tool like the GSED may not capture this full complexity. Differences between medium and high SES children appear, on average, much smaller, and were primarily visible in the preschool period. These findings align with global evidence on the developmental effects of early socioeconomic disadvantage, while also highlighting patterns unique to Tanzania and sub-Saharan Africa, as explored further in this discussion.

These observed patterns reflect the result of multiple, intersecting causal pathways operating across biological (e.g., child birthweight, nutrition, infectious diseases, genetics), environmental (e.g., home environment, play material, healthcare access), and psychosocial factors (e.g., parental mental health, parent-child interactions, cognitive stimulation, learning opportunities). The results presented here are not meant to provide causal effect sizes, but simply to characterize the magnitude of developmental differences at a population level [19,68–70].

Educational access is one of pathway where these inequalities become clearly visible., as we found that children aged 6–8 from high SES households show higher primary school enrollment than their counterparts from low SES families, highlighting a significant educational disparity. This is consistent with other research conducted in low- and middle-income nations including Nigeria, Malawi, and Uganda. [71–77]. The observed disparity arises often as a result of unequal access to crucial resources, as high SES families can more readily afford school fees, books, uniforms, and transportation costs, particularly when schools are located far from their homes, even in countries with universal primary education policies like Tanzania [75].

Meanwhile, due to the direct and opportunity costs of expecting children to assist at home, on the family farm, or by earning additional money through child work, low SES frequently delays or precludes enrollment.[74,75,78]. The Tanzanian pattern reflects a bigger educational inequality across sub-Saharan Africa with delayed or absent school entry limiting cognitive stimulation for low-SES children and prolonging exposure to adverse home environments, negatively affecting early neurodevelopment. These results reinforce the urgent need for early, targeted support to promote school readiness and enrollment among low-resource families.

Beyond educational access specifically, the data reveals a wider gradient of SES-linked neurodevelopmental disadvantage that persists across childhood. Our study revealed that socioeconomic disparities in neurodevelopmental scores appear to emerge after the first two years of life, with children in the highest SES tertile consistently achieving higher mean scores than those in the low SES tertile across the subsequent age span studied. These results support well-established links between SES and cognitive development in children [2,8,14,69]. The Young Lives multi-country cohort, which tracked children across Ethiopia, India, Peru, and Vietnam, reported similar trends in SES gradients in cognitive composite scores [79]. Similarly, data from 95 national surveys conducted in LMICs showed that early-life poverty had a detrimental impact on children’s cognitive development [80]. The similarity of our findings with those from comparable LMIC settings strengthens confidence in the validity of our findings and suggests that the SES-neurodevelopment relationship in Tanzania follows regional patterns.

Importantly, our study enhances this understanding by detailing these trends within a rural Tanzanian context, emphasizing the distinct trajectory of disparity emergence. We hypothesize that potential mechanisms for the observed disparities include lack of cognitive stimulation, poor nutrition, and psychosocial stressors associated with low SES. These factors are well-documented to impact neurodevelopmental trajectories as children mature and cognitive demands rise [8,43,69,81–83].

To understand how SES disparities evolve during various stages of childhood, we hypothesized that the transition into preschool and formal schooling might mitigate some of the initial socioeconomic gradients. However, our findings revealed the opposite: In our sample, SES disadvantages become larger during the preschool period, and this effect continued into the formal school years. This finding contrasts with studies showing disparities in neurocognition and neural structures existing within the first 2 years of life [14]. This difference may be explained by the nature of early childhood development, where certain fundamental neurodevelopmental competencies are still emerging and caregiving practices (i.e., feeding, comforting, basic parental interaction) might be more universally sufficient across SES for basic development, potentially buffering the full impact of SES disadvantages in the youngest ages [81].

Additionally, while Noble et al [14] used tools that focus on specific cognitive areas like language and memory, which are sensitive to very early environmental differences, our study used a broader, more comprehensive neurodevelopmental tool in that age group, i.e., GSED. This wider measure may not pick up on the subtle, early disparities but instead captures the larger, more generalized gap that becomes significant and measurable after age 2. Also, contextual factors and exposure duration might also explain the differences observed. The widening of socioeconomic disparities in neurodevelopment from the preschool to school-age years is consistent with a cumulative risk framework [84]. This approach implies that the harmful impacts of poverty are not static from birth but rather compound throughout life as risks increase. This pattern is shown in longitudinal data from the U.S. Fragile Families and Child Wellbeing Study, which found that socioeconomic cognitive gaps, initially modest in infancy, expanded significantly between ages 3 and 9, particularly within the domains of language and executive function [85].

Complementing this cumulative risk perspective, as children grow, the quality of their home environment and the nature of caregiver interactions become crucial for developing advanced cognitive skills. Children from low SES households often face cumulative disadvantages due to fewer enriching experiences and less cognitive stimulation [14,22,36,37,81,86]. This is regarded as transactional model, and offers more direct explanation of how poverty leads to growing developmental differences through the everyday experiences children encounter at home and in their caregiving relationships [87].

Furthermore, while school quality differences can mitigate SES gaps during preschool or school entry, the absence of such mitigation in our study suggests that this context’s educational environment may not be fully addressing existing disparities. Some potential examples of factors associated with this lack of mitigation could include aspects of school quality (e.g., overall school environment, staff-child relationships, curriculum) or inconsistent attendance, which could contribute to or fail to reduce neurodevelopment disparities in this setting [88,89].

Our analysis found no statistically significant differences in neurodevelopment between middle- and high-SES children at any age. This suggests that while SES impacts neurocognitive function across the population, the strongest effects are seen in the lowest SES groups. Research from international cohorts in the US and Europe also demonstrates that the relationship between family income and a child’s cognitive development is non-linear, following a notable “plateau” pattern. While cognitive performance improves sharply as families rise out of poverty, this benefit levels off once a middle-income threshold is reached [90,91]. Evidence from the Young Lives cohort in LMICs show similar findings, a non-linear relationship where the cognitive benefits for children begin to taper off. In these settings, once a family surpasses a moderate wealth threshold, further improvements in their SES result in increasingly smaller gains in a child’s cognitive development [79,92].

Several factors may explain this: reduced environmental variability among middle- and high SES families might limit our ability to detect subtle cognitive differences; therefore, additional wealth beyond a middle-SES level may not significantly benefit neurodevelopment; or our assessment tools, while reliable and valid, may lack the sensitivity to detect small, domain-specific cognitive gains associated with higher affluence beyond middle-SES. The lack of significant disparity doesn’t contradict the established SES gradient in child neurodevelopment; rather, it highlights a potential plateau effect once basic needs are met. The consistency of these findings across different global settings indicates that the observed plateau is a real phenomenon. It also highlights a critical economic threshold where poverty causes significant developmental harm, while wealth beyond that point produces diminishing developmental benefits. Regardless, this has fundamental policy implications: interventions aimed at aiding families from resource-poor to middle SES may produce the highest neurodevelopmental returns, while additional resource investments among already advantaged households may offer less cognitive advantages among children in those households.

This study reveals how household wealth is associated with children across developmental stages. Our study used random sampling to reduce sampling bias, and we statistically adjusted for enumerator effects in our models to mitigate potential interviewer-related bias. Nonetheless, the influence of other potential biases, such as retrospective recall or social desirability bias, cannot be entirely excluded. Our study used asset ownership and did not account for other factors like age of household head, caregiver education level, number of household members earning an income, and income to household size ratio, which are all linked to household wealth, hence, this technique offers only a partial view of the multi-layered nature of low SES, inequity, and inequality [65]. Lastly, unassessed factors, such as genetic predispositions, environmental effects, or access to early childhood education and healthcare services, could have confounded the relationship between SES and neurodevelopmental outcomes.

While these limitations are characteristic of neurodevelopmental research in LMICs, they do not undermine the study’s contributions; instead, they establish a clear trajectory for future research. Subsequent studies in Tanzania should employ multidimensional socioeconomic measures, using asset-based wealth measures alongside caregiver education, income stability, and neighborhood-level indicators to pinpoint which specific aspects of disadvantage most directly impact neurodevelopmental risks.Despite the limitations, we believe our study offers substantial strength. To the best of our knowledge, this is the first study to examine socioeconomic disparities in children’s neurodevelopment across a broad developmental period, i.e., 0.5 to 10 years. Additionally, our use of a population-based sample drawn from diverse households participating in the CHILD parent study, across both rural and semi-urban settings, enhances the internal validity and relevance of these findings within similar low-resource environments. By demonstrating that disparities are negligible in the first two years, widening during the preschool period, and persisting into formal schooling, our study generates a developmentally grounded evidence base that can directly inform the timing, targeting, and design of intervention programs in this setting.

Furthermore, the usage of a locally validated and adapted cognitive assessment battery and a PCA-based wealth index allowed us to capture contextually relevant measures of neurodevelopment and household SES. However, generalizability beyond the study setting may be limited due to the exclusion of children outside the formal health system, who might be at higher risk of low SES-related exposures. Additionally, cultural adaptations of assessment tools, though necessary, may also affect comparability with other settings. Nonetheless, our findings highlight patterns relevant to similar populations across sub-Saharan Africa and identify key areas for targeted early interventions.

## 5. Conclusion and recommendations

This study highlights the persistent and widening neurodevelopment disparities among children from low-SES households in Tanzania, with significant effects seen as children transition to the preschool period and persisting despite school exposure. Therefore, interventions aimed at reducing these disparities must begin early and prioritize the most disadvantaged households. The developmental window of greatest importance, based on our findings, is the period between 24 and 72 months (preschool period) during which SES-related gaps first become measurable and substantial. Early Childhood Development (ECD) Programs implementing or expanding community-based structured ECD programs that not only provide direct stimulation for children but also heavily involve parents and caregivers in learning activities and support should be implemented. This helps build a strong foundation before the cognitive gaps become pronounced. Additionally, developing and implementing specific programs aimed at bridging the cognitive gap for children from the poorest households before and during their transition to preschool and early primary school. However, improving school attendance alone will not close these gaps. Quality improvement in primary school environments, particularly in terms of teacher training, class sizes, instructional resources, and curriculum alignment with developmental needs, is an essential complement to any intervention.

We also recommend implementing broader socioeconomic interventions that alleviate household low SES while specifically incentivizing and supporting children’s educational and developmental progress, especially during the critical preschool and early school-age years, to address the observed gap. Conditional cash transfer programs that link income support to behavioral conditions such as health check-up attendance, school enrollment, and participation in parenting programs could be beneficial. Lastly, the neurodevelopment assessment tools employed in this study could be employed in ongoing surveillances and to effectively monitor the impact of social, educational, and health interventions as they are rolled out.

## Supporting information

S1 Table Association between overall neurodevelopmental scores and socioeconomic status (SES) tertiles. (DOCX)

S2 Table Predicted Age-Standardized Neurodevelopment Scores by Socioeconomic Status (SES) Tertile within Childhood Exposure Groups. (DOCX)

S3 Table Association between children’s age-residualized neurodevelopmental scores, family socioeconomic status (SES) tertiles, and childhood age periods. (DOCX)

S4 File Inclusivity in global research questionnaire. (DOCX)

S1 Data Cleaned, de-identified minimal dataset underlying the study findings. (CSV)

S1 Text Stata analysis script containing code for data management, multiple imputation, and multivariable regression models. (TXT)

## Abbreviations

AIDS: Acquired Immuno Deficiency Syndrome
AL: Artemether Lumefantrine
ALSPAC: Avon Longitudinal Study of Parents and Children
CHILD: Child Health and Infection with Low Density Malaria
CI: Confidence Interval
CREDI: Caregiver Reported Early Development Instrument
EACAB: East African Cognitive Assessment Battery
ECD: Early Childhood Development
EDC: Electronic Data Capture
GSED: Global Scales for Early Development
HIV: Human Immunodeficiency Virus
HLE: Home Literacy Environment
IDELA: International Development and Early Learning Assessment
IHI: Ifakara Health Institute
IQR: Interquartile Range
LMIC: Low- and Middle-Income Countries
MDAT: Malawi Developmental Assessment Tool
NIMR: National Institute of Medical Research
NIH: National Institutes of Health
ODK: Open Data Kit
PCA: Principal Component Analysis
PO-RALG: President’s Office, Regional Administration and Local Government, Tanzania
SD: Standard Deviation
SES: Socioeconomic Status
SSA: Sub-Saharan Africa
TEA-Ch: Test of Everyday Attention for Children
