# Supplementary material for: Socioeconomic disparities in children’s neurodevelopment before and during primary education: Evidence from Bagamoyo, Tanzania
Source: PLoS One. 2026 Jul 27;21(7):e0354139. doi: 10.1371/journal.pone.0354139 (PMC13405314; doi:10.1371/journal.pone.0354139)
Supplement: S1 Table — (DOCX) [file pone.0354139.s001.docx]

**Table1. Association between overall neurodevelopmental scores and socioeconomic status (SES) tertiles.**

| **Outcome** | **Variable** | **Coefficient** | **P-value** | **95% CI Lower** | **95%CI Upper** |
| --- | --- | --- | --- | --- | --- |
| **Overall Neuro score** | **SES tertiles** |  |  |  |  |
| **Composite Z-score**  **(0.5-10 years)** | Low* | -0.13 | 0.051 | -0.26 | 0.00 |
|  | Middle | 0.15 | 0.150 | -0.05 | 0.35 |
|  | High | 0.25 | 0.011 | 0.06 | 0.44 |
|  |  |  |  |  |  |

This table presents the regression results for the association between the overall neurodevelopmental composite z-score and SES tertiles, with Low SES families as the reference category.

*Reference category
