# Supplementary material for: Socioeconomic disparities in children’s neurodevelopment before and during primary education: Evidence from Bagamoyo, Tanzania
Source: PLoS One. 2026 Jul 27;21(7):e0354139. doi: 10.1371/journal.pone.0354139 (PMC13405314; doi:10.1371/journal.pone.0354139)
Supplement: S2 Table — (DOCX) [file pone.0354139.s002.docx]

**Table 2. Predicted Age-Standardized Neurodevelopment Scores by Socioeconomic Status (SES) Tertile within Childhood Exposure Groups.**

| **Childhood period # Socioeconomic Tertile** | **Coefficient** | **P-Value** | **95%CI Lower** | **95% CI Higher** |
| --- | --- | --- | --- | --- |
| Childhood Age # Resource-poor SES | -0.02 | 0.796 | -0.16 | 0.12 |
| Childhood Age # Middle SES | -0.03 | 0.726 | -0.21 | 0.15 |
| Childhood Age # High SES | 0.05 | 0.566 | -0.11 | 0.20 |
| Preschool Age # Resource-poor SES | -0.19 | 0.055 | -0.38 | 0.00 |
| Preschool Age # Middle SES | 0.02 | 0.881 | -0.19 | 0.22 |
| Preschool Age # High SES | 0.21 | 0.053* | 0.00 | 0.41 |
| School Age # Resource-poor SES | -0.24 | 0.032* | -0.46 | -0.02 |
| School Age # Middle SES | 0.13 | 0.306 | -0.12 | 0.38 |
| School Age # High SES | 0.17 | 0.172 | -0.07 | 0.41 |

This table displays the regression coefficients, confidence intervals, and p-values for the predicted neurodevelopment scores by SES tertile across different childhood age periods.

*P-Value ≤ 0.05

# Interaction term
