# Supplementary material for: Socioeconomic disparities in children’s neurodevelopment before and during primary education: Evidence from Bagamoyo, Tanzania
Source: PLoS One. 2026 Jul 27;21(7):e0354139. doi: 10.1371/journal.pone.0354139 (PMC13405314; doi:10.1371/journal.pone.0354139)
Supplement: S3 Table — (DOCX) [file pone.0354139.s003.docx]

**Table 3. Association between children's age-residualized neurodevelopmental scores, family socioeconomic status (SES) tertiles, and childhood age periods.**

| **Age-residualized neurodevelopment scores** | **Coefficient** | **p-value** | **95% CI Lower** | **95% CI Upper** |
| --- | --- | --- | --- | --- |
| **Socioeconomic Tertile (SES)** |  |  |  |  |
| Resource-Poor | -0.06 | 0.550 | -0.27 | 0.15 |
| Middle | -0.08 | 0.524 | -0.32 | 0.16 |
| High | Reference |  |  |  |
|  |  |  |  |  |
| **Period** |  |  |  |  |
| Preschool | 0.16 | 0.220 | -0.10 | 0.42 |
| School | 0.12 | 0.399 | -0.16 | 0.41 |
| Early childhood | Reference |  |  |  |
|  |  |  |  |  |
| **SES # Period** |  |  |  |  |
| Low x Preschool | -0.33 | 0.065 | -0.68 | 0.02 |
| Low x School | -0.34 | 0.080 | -0.73 | 0.04 |
| Middle x Preschool | -0.11 | 0.558 | -0.49 | 0.27 |
| Middle x School | 0.04 | 0.851 | -0.38 | 0.46 |
|  |  |  |  |  |
| Cons | 0.05 | 0.566 | -0.11 | 0.20 |

This table presents the regression coefficients, p-values, and confidence intervals for the main effects of socioeconomic tertile and age period, as well as their interaction effects, on children's neurodevelopmental scores.

# Interaction term
